# Drug Resistance Mechanisms in Bacteria Causing Sexually Transmitted Diseases and Associated with Vaginosis

**DOI:** 10.3389/fmicb.2016.00747

**Published:** 2016-05-18

**Authors:** Boris Shaskolskiy, Ekaterina Dementieva, Arvo Leinsoo, Anastassia Runina, Denis Vorobyev, Xenia Plakhova, Alexey Kubanov, Dmitrii Deryabin, Dmitry Gryadunov

**Affiliations:** ^1^Laboratory for Molecular Diagnostics Technologies, Engelhardt Institute of Molecular Biology, Russian Academy of SciencesMoscow, Russia; ^2^State Research Center of Dermatovenerology and Cosmetology of the Russian Ministry of HealthMoscow, Russia

**Keywords:** human reproductive system, sexually transmitted diseases, bacterial vaginosis, antimicrobials, antimicrobial resistance

## Abstract

Here, we review sexually transmitted diseases (STDs) caused by pathogenic bacteria and vaginal infections which result from an overgrowth of opportunistic bacterial microflora. First, we describe the STDs, the corresponding pathogens and the antimicrobials used for their treatment. In addition to the well-known diseases caused by single pathogens (i.e., syphilis, gonococcal infections, and chlamydiosis), we consider polymicrobial reproductive tract infections (especially those that are difficult to effectively clinically manage). Then, we summarize the biochemical mechanisms that lead to antimicrobial resistance and the most recent data on the emergence of drug resistance in STD pathogens and bacteria associated with vaginosis. A large amount of research performed in the last 10–15 years has shed light on the enormous diversity of mechanisms of resistance developed by bacteria. A detailed understanding of the mechanisms of antimicrobials action and the emergence of resistance is necessary to modify existing drugs and to develop new ones directed against new targets.

## Introduction

The infections of human reproductive system include the sexually transmitted diseases (STDs) that are defined as infections that spread primarily through person-to-person sexual contact, and non-STDs which are endogenous infections of the genital organs such as bacterial vaginosis (BV). Both STDs and non-STDs are the major concern for public health systems worldwide.

According to the WHO, each year there are approximately half a billion new cases of STDs worldwide (WHO, 2014a), of which 105.7 million are chlamydial infections, 106.1 million are gonorrhea, and 10.6 million are syphilis (WHO, 2013). STDs lead to a decrease in fertility and are harmful to the offspring. Up to 40% of women with untreated gonococcal and chlamydial infections develop inflammatory diseases of the pelvic organs that lead to infertility in 25% of cases; pregnancy in women with the untreated early forms of syphilis results in stillbirth in 25% of cases and neonatal death in 14% (CDC, 2015). Obviously, the timely detection of STDs and adequate antimicrobial therapy are crucial for the successful treatment and prevention of the spread of the disease.

STDs are often polymicrobial and are caused not only by obligate pathogens (*Neisseria gonorrhoeae, Chlamydia trachomatis, Treponema pallidum*, and *Mycoplasma genitalium*) but also by a variety of opportunistic microorganisms which are mostly not sexually transmitted and can occur without sexual relationship. The polymicrobial nature of the reproductive tract pathologies modifies the clinical pattern of the disease, aggravates the inflammatory process, and reduces the efficacy of the determination of their etiologies, which can result in inadequate treatment (Josey and Schwebke, 2008).

A major worldwide problem is the increase in drug resistance of pathogens, which makes it difficult to select proper treatments. In April 2014, the WHO published the report “Antimicrobial resistance: A global report on epidemiological surveillance” (WHO, 2014b). The report emphasized the threat of an increase in the resistance of many pathogens. The greatest concern in addition to tuberculosis, malaria, and staphylococcal infection is the emergence of *N. gonorrhoeae* strains with reduced susceptibility to third-generation cephalosporins. Thus, elucidating the etiology of infections and the concurrent inflammatory processes of the urogenital tract and developing successful therapies require the detection of the primary infectious agents in clinical specimens, the simultaneous identification of a large number of other opportunistic pathogens and an analysis of their susceptibility to common antimicrobial drugs.

This review focuses on the characterization of the most common sexually transmitted bacterial pathogens (excluding viral, fungal, and parasitic infections) and non-STD infections which result from an overgrowth of opportunistic bacterial microflora, and discusses the mechanisms and molecular determinants of their drug resistance.

## Brief description of the most common bacterial infection of the human reproductive system and the drugs used to treat them

### Syphilis

This disease is caused by the sexually transmitted, Gram-negative, spirochetal bacterium *T. pallidum* and is characterized by lesions of the skin, mucous membranes, nervous system, internal organs, and musculoskeletal system. Syphilis can cause intrauterine infections of the fetus. The disease can occur in both symptomatic and latent forms. Despite significant achievements in the fight against this disease, syphilis remains a challenge for treatment and public health care due to the variations in the course of the disease and the diversity of its clinical manifestations. Syphilis is treated with benzathine penicillin G, tetracycline and its derivative doxycycline, macrolides (e.g., erythromycin and azithromycin), and cephalosporins (e.g., the third generation drug ceftriaxone). The main drug of choice for syphilis treatment in the 2015 CDC recommendations is benzathine penicillin G (Workowski and Bolan, 2015). Patients allergic to penicillin are treated with the other antibiotics mentioned above. In clinical trials, some antibiotics (e.g., azithromycin) proved to be more effective than penicillin G for the treatment of early syphilis (Bai et al., 2008).

### Gonococcal infection

Gonorrhea is one of the most common STDs and is caused by the Gram-negative diplococci *N. gonorrhoeae*. Health complications resulting from the gonococcus disease occur mainly in women and are largely attributed to the predominately asymptomatic nature of lower genital tract, i.e., cervical, infection. Untreated, subclinical infection of the cervix can lead to upper genital tract involvement (e.g., salpingitis) and, potentially, to infertility. The infection primarily affects the genitals, including the cylindrical and glandular epithelium. Although gonococcal vaginitis develops in female children in which menarche has not yet occurred, keratinization occurring with menarche prevents gonococcal vaginitis in the adult female (Edwards and Butler, 2011).

Gonococcal infection is usually treated with ciprofloxacin, ofloxacin, spectinomycin, and β-lactam antibiotics; currently, the most effective recommended drugs are ceftriaxone and azithromycin (Workowski and Bolan, 2015). The widespread occurrence of penicillin-resistant gonorrhea has led to a decrease in the use of first and second generation β-lactams, protected penicillins (clavulanate-potentiated amoxicillin or ticarcillin-clavulanate), and cephalosporins in clinical practice. Ceftriaxone belonging to cephalosporin drugs, that are more resistant to β-lactamases, remains widely used.

Spectinomycin, from the aminocyclitol class of drugs closely related to the aminoglycosides, since 1960s was used for specific treatment of gonorrhea. It was useful in an alternative therapy for gonorrhea resistant to antibiotics of the other groups or in patients allergic to β-lactams. After 10–20 years of treatment, spectinomycin-resistant gonococcal strains were reported in Netherlands, South Korea, United Kingdom, and USA (Unemo and Shafer, 2014). There is a consistent trend of increasing resistance of gonococci to spectinomycin in Russia (up to 15% in 2010) (Kubanova et al., 2010, 2014). Spectinomycin is not used now as a first-line drug for gonorrhea therapy in many countries.

### Chlamydiosis

The infection is caused by the Gram-negative intracellular bacterium *C. trachomatis*. There are 15 identified serovars of *C. trachomatis*. Serotypes Ab, B, Ba, and C are pathogens that cause trachoma, serovars D, E, F, G, H, I, J, and K cause urogenital chlamydiosis, urethritis, prostatitis, vaginitis, and chlamydial cervicitis, and serovars L1, L2, and L3 cause lymphogranuloma venereum and genital ulcers (Workowski and Bolan, 2015). The socio-economic significance of urogenital chlamydial infection is due not only to the high rate of disease but also to a significant percentage of complications, especially the development of secondary infertility in men and women (Vasilevsky et al., 2014).

The drugs of choice for the treatment of chlamydial infections are tetracyclines (doxycycline) and macrolides (josamycin and azithromycin); alternatively, fluoroquinolones (levofloxacin and ofloxacin) can be used (Workowski and Bolan, 2015). Despite the availability of a whole range of drugs against chlamydia, its treatment is not always successful due to the presence of co-infections with the causative agent of urogenital ureaplasmosis (*Ureaplasma urealyticum*). Thus, adequate therapy requires the accurate identification of additional microorganisms associated with the chlamydia infection and the simultaneous treatment of opportunistic infections.

### Mycoplasmosis and ureaplasmosis

The diseases are caused by opportunistic microorganisms of the genus *Mycoplasma* (*M. genitalium* and *Mycoplasma hominis*) and *Ureaplasma*. The latter is classified in a separate genus due to its ability to break down urea. Researchers have identified two species of *Ureaplasma* (*U. parvum* and *U. urealyticum*) that are further classified into 14 serovars. Mycoplasma and ureaplasma are detected in more than 40% of patients with inflammatory diseases of the urogenital system; three species have the highest clinical significance (*M. genitalium, U. urealyticum*, and *M. hominis*). Mycoplasma and ureaplasma can cause urethritis in both sexes and probably cervicitis, cystitis, and pregnancy complications including post-partum period and post-abortion complications (Larsen and Hwang, 2010). The drugs of choice for the treatment of infections are macrolides (azithromycin and josamycin) and tetracyclines (doxycycline). Alternative medications are fluoroquinolones (levofloxacin and ofloxacin) (Workowski and Bolan, 2015).

### Bacterial vaginosis (BV)

BV is an infectious non-inflammatory disease of a polymicrobial nature that is predominantly detected in women of reproductive age. The disease is not immediately life-threatening, but it is a risk factor for pregnancy complications and pelvic inflammatory diseases. Moreover, BV is associated with increased risk for acquiring sexually transmitted infections (Martin, 2012). For example, BV can increase a woman's risk of acquiring *N. gonorrhoeae* (gonococcal infection hazard ratio: 1.7; 95% CI: 1.1–2.6), *C. trachomatis* (chlamydial infection odds ratio: 3.4; 95% CI: 1.5–7.8), and *Trichomonas* (trichomonal genital infection hazard ratio: 1.8; 95% CI: 1.3–2.4) (Margolis and Fredricks, 2015).

BV occurs as the result of a drastic imbalance in the normal vaginal microflora rather than as an invasion of a foreign pathogen. In this case, the normal protective lactobacilli are replaced by high quantities of anaerobes (Turovskiy et al., 2012; Margolis and Fredricks, 2015). Among the most commonly detected causative agents of the disease are the microaerophilic bacteria *Gardnerella vaginalis*, obligate anaerobic Gram-positive bacteria (*Mobiluncus* spp. and *Peptostreptococcus* spp.), and the facultative anaerobic bacteria *Atopobium vaginae*. Additionally, the obligate anaerobic Gram-negative bacteria *Prevotella* spp.*, Bacteroides* spp., and *Fusobacterium* spp. are sometimes present in clinical samples (Verhelst et al., 2004; Menard, 2011). The list of bacteria that have been shown to be associated with vaginosis is constantly widening (i.e., *Dialiester, Prevotella*, and *Megasphaera*) (Margolis and Fredricks, 2015).

*G. vaginalis* bacteria are commonly detected at moderately high concentrations in BV-positive women but their presence alone is not specific for BV (Kalra et al., 2007). The frequent detection of genital mycoplasmas together with anaerobic microflora is due to the ability of *G. vaginalis* to secrete succinic acid, which is used by other microorganisms. In turn, ureaplasma and mycoplasma, which actively consume oxygen during their metabolism, stimulate the proliferation of anaerobic bacteria (Africa et al., 2014). Agents active against *G. vaginalis* include metronidazole and clindamycin.

The proportion of *A. vaginae* (another causative agent of vaginitis) ranges from 50 to 95%. This Gram-positive anaerobic bacillus from the *Coriobacteriaceae* family produces organic acids and can be found together with *G. vaginalis* within the layer of microorganisms on the surface of the vaginal mucosa in bacterial vaginosis patients (Verhelst et al., 2004). The dominant growth of *A. vaginae* is also treated with metronidazole and clindamycin.

Mobiluncus is a motile anaerobic microorganism belonging to the family *Actinomycetaceae*. Two species of the genus *Mobiluncus* are usually found in the human genitourinary tract (*M. curtisii* and *M. mulieris*). Both bacteria are susceptible to β-lactam antibiotics, glycopeptide drugs, clindamycin, chloramphenicol, and rifampicin and are primarily resistant to metronidazole (Spiegel, 1987). Currently, infections caused by *Mobiluncus* spp. are commonly treated with clindamycin (Verstraelen and Verhelst, 2009).

*Bacteroides fragilis* is a Gram-negative anaerobic bacterium that is a constituent of the normal microflora of the vagina and vulva. *B. fragilis* may cause pyelonephritis, cystitis, urethritis, prostatitis, and other infectious diseases of the genitourinary system in both sexes. The main drug for the treatment of infections caused by *B. fragilis* is metronidazole (Löfmark et al., 2010); alternative treatments include β-lactam antibiotics in combination with β-lactamase inhibitors, clindamycin and chloramphenicol.

Taking into account the leading role of obligate anaerobic bacteria in BV, the drugs of choice for BV treatment are those with anti-anaerobic activity (e.g., clindamycin and 5-nitroimidazole derivatives). Notably, effective BV treatment must be based on a differential diagnosis with other urogenital diseases caused by pathogens (*N. gonorrhoeae, C. trachomatis, Trichomonas vaginalis*, and *M. genitalium*) (Menard, 2011; Workowski and Bolan, 2015).

## Drug resistance of STD pathogens

The continuously increasing resistance of STD pathogens to antimicrobial agents is a worldwide problem. Antibiotics that have lost their effectiveness are replaced by new drugs, but new strains appear with new determinants of resistance; this issue applies to all classes of drugs. The increase in the resistance and diversity of the drugs used in clinical practice leads to the emergence of bacteria with multidrug resistance (MDR). Some strains with MDR have the ability to spread rapidly (so-called high-risk clones). Some pathogens acquire simultaneous resistance to most drugs developed for their treatment (extensively drug-resistant pathogens) or even to all drugs (pandrug resistance) (Unemo and Nicholas, 2012; Rossolini et al., 2014). Currently, multidrug-resistant isolates have been identified for both Gram-positive and Gram-negative species, including *N. gonorrhoeae*.

### T. pallidum

Although diagnostic tests for syphilis and antibiotic therapy are now available, the disease remains endemic in many developing countries. Widespread syphilis epidemics occurred in Russia in the 1990s (Stamm, 2010) and more recently in China (Tucker and Cohen, 2011). A recent increase in syphilis rates in women and infants in the USA has also been described (CDC, 2015).

Although penicillins have been used for the treatment of syphilis for 70 years, no natural penicillin-resistant forms have been found. To date, no clinically proven cases of treatment failure using penicillin-related drugs have been described in patients with syphilis. However, the possibility of the emergence of acquired resistance is still discussable (Stamm, 2010). Recent analysis of *T. pallidum* resistance to antibiotics in the Russian Federation revealed no resistance to β-lactam antibiotics. Specifically, no meaningful mutations were found in the *tp47* and *tromp* genes encoding the targets of β-lactams (Kubanova et al., 2013).

Currently, there is no documented resistance of *T. pallidum* to the tetracyclines. Clinically significant resistance of *T. pallidum* to macrolides (a second-line alternative to penicillin) has been demonstrated by many authors, and macrolide-resistant strains are now prevalent in several developed countries (Stamm, 2010). Clinical cases of *T. pallidum* resistance to macrolides (particularly erythromycin; Stamm and Bergen, 2000), azithromycin (Katz and Klausner, 2008; Stamm, 2010), clarithromycin (Stamm, 2010), and spiramycin (Matějková et al., 2009) have been reported. There was a report concerning treatment failure with clindamycin but these data were not confirmed and *T. pallidum* appeared to have intrinsic resistance to this drug (Stamm, 2010).

The first strain resistant to erythromycin and azithromycin was isolated in 1977 (Street strain 14) (Stamm, 2012). Sequencing of the *T. pallidum* genome has shown that it lacks the genetic elements responsible for horizontal gene transfer (plasmids and transposons); thus, it was concluded that the resistance to macrolides in Street strain 14 emerged endogenously by a spontaneous chromosomal mutation in the 23S rRNA gene. The mutations responsible for macrolide resistance were shown to be A2058G and A2059G (23S rRNA). Mutation A2058G was reported in several areas of the USA, Canada, Europe, and China (Stamm, 2012), whereas the replacement of A2059G was less common but was not geographically isolated (Matějková et al., 2009; Cruz et al., 2013). There were reports concerning patients with *T. pallidum* bearing the A2059G mutation in the Czech Republic (Matějková et al., 2009), Columbia (Cruz et al., 2013), and the UK (Tipple et al., 2011). The proportion of isolates resistant to macrolides has been growing, indicating a need for further drug development and surveillance for resistance in *T. pallidum* (Tipple et al., 2011).

### N. gonorrhoeae

The pathogen *N. gonorrhoeae* is characterized by an extraordinary ability to develop resistance to clinically used antimicrobial drugs within 10–20 years (Unemo and Nicholas, 2012). *N. gonorrhoeae* is able to quickly accumulate mutations and acquire resistance to drugs, including multiple drug resistance. There are now strains of *N. gonorrhoeae* that are resistant to all major drugs used to treat gonorrhea, including β-lactams, fluoroquinolones, macrolides, tetracyclines, spectinomycin, cephalosporins, and azithromycin. The spread of super-resistant forms of the gonorrhea pathogen is a major concern and requires the strengthening of control on a global scale (WHO, 2014b). Given the rate of increase of *N. gonorrhoeae* resistance to other drugs, some cases of gonorrhea may become incurable by 2021 because no vaccines or new drugs are being developed (Unemo, 2015). Unemo and his coworkers in Sweden and the WHO report have repeatedly stressed that the situation with drug-resistant gonorrhea can spiral out of control; thus, it is extremely urgent to develop rapid genetic analysis methods to monitor the resistance patterns of the pathogen worldwide and to apply these data to the treatment of patients (Unemo et al., 2014; WHO, 2014b).

**Figure 1:**
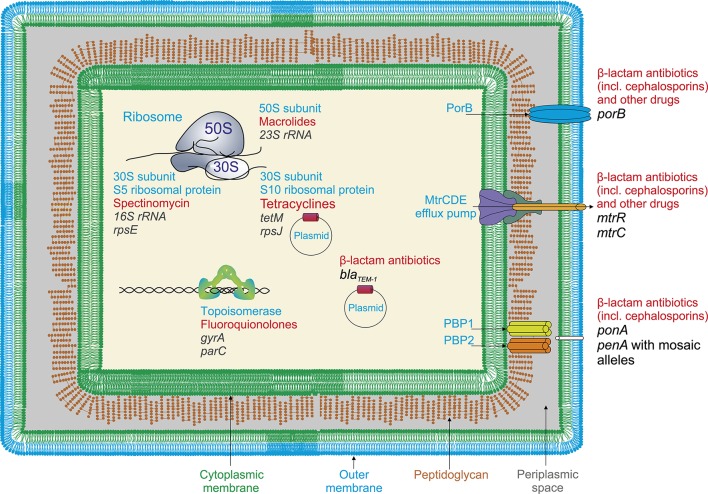
**Resistance determinans in ***N. gonorrhoeae*** for widely used antimicrobial drugs (a general scheme)**. Resistance to β**-lactam antibiotics** (penicillins) appears due to the presence of *bla*_*TEM*−1_plasmid, encoding β-lactamase, and mutations in penicillin-binding proteins PBP1 and PBP2 (*penA* and *ponA* genes); **cephalosporins**, mutations in plasmid genes encoding β-lactamase and *penA* gene involving appearance of mosaic *penA* alleles with up to 70 amino acid alterations; **fluoroquinolones**, mutations in *gyrA* and *parC* genes encoding DNA gyrase and topoisomerase; **tetracyclines**, the presence of *tetM*-encoding plasmids and mutations in *rpsJ* gene encoding S10 ribosomal protein (ribosome 30S subunit); **macrolides**, mutations in 23S RNA genes encoding peptidyltransferases, rRNA-methylases; **spectinomycin**, mutations in *rpsE* gene encoding S5 ribosomal protein (23S rRNA); **various classes of drugs**, structural changes in major outer membrane porin protein (PorB) resulting in reducing of membrane permeability; and also mutations in efflux pump genes, particularly, in *mtrR* genes in the promoter or coding region, causing increased efflux.

Gonococci use most of the known mechanisms to acquire resistance: the inactivation of the antibiotic, alteration of the drug binding sites, reduction of membrane permeability, and increased drug efflux (Figure 1). The bacteria acquire these new characteristics via chromosomal mutations, plasmids carrying the determinants of resistance (i.e., *TEM-1, TEM-135*-encoding plasmids for resistance to penicillins, *TetM*-encoding plasmids for tetracycline resistance), and horizontal gene transfer from other species, especially from other species of *Neisseria*. Mutations altering the membrane permeability and increasing the activity of the efflux pumps are particularly efficient in the emergence of in *N. gonorrhoeae* because both mechanisms affect a wide variety of drugs.

When the penicillin treatment regimen was initiated in the 1940s, this drug was highly effective against gonococci. However, within the next 10–15 years a gradual decrease in penicillin susceptibility was observed; this decrease was mainly due to the sequential accumulation of chromosomal mutations in the bacteria. As the result, strains with MIC for penicillin as high as 4.0 mg/L were formed. Over the next few years, the same process was repeated with erythromycin, tetracycline, and azithromycin (Unemo and Shafer, 2014).

There are several mechanisms that explain the resistance of *N. gonorrhoeae* to β-lactam antibiotics. First, the bacteria produce TEM β-lactamases using a plasmid gene (*N. gonorrhoeae* does not produce chromosomally-encoded beta-lactamases). Because the gonococcal β-lactamase encoded by the plasmid gene *bla*_*TEM*−1_ is not able to destroy cephalosporins, these antibiotics have taken a leading role in the treatment of gonorrhea (Klausner and Kerndt, 2013). Mutations that decrease the affinity of the corresponding penicillin-binding proteins PBP1 and PBP2 to penicillins have been identified in the chromosomal genes *ponA* and *penA*. The main mutation in the *ponA* gene leading to the development of resistant phenotype is L421P (Lindberg et al., 2007; Endimiani et al., 2014), whereas an insertion in the *penA* gene leads to the appearance of D345 (Lindberg et al., 2007; Jeverica et al., 2014). The insertion of an extra codon for D345 in the wild-type *penA* gene results in a four-fold reduction in the susceptibility of gonococci to penicillin (Spratt, 1988).

In addition to the various mechanisms of penicillin resistance of *N. gonorrhoeae* described above, a mutation in the *pilQ2* gene (E666K) was found to increase resistance to penicillin *in vitro*. E666K works in combination with mutations in the *penA, mtrR*, and *penB* genes (Zhao et al., 2005; Helm et al., 2007). Because mutations in the *pilQ2* gene disrupt the formation of pili, which are required for the pathogenicity of gonococci, it is unlikely that similar genotypes could be found in clinical specimens.

During the past few years, the rate of gonococcal strains resistant to penicillin in the Russian Federation has varied between 9.6 and 13.2% (Kubanova A. et al., 2014). Globally, the proportion of resistant *N. gonorrhoeae* strains has widely varied: 81% in China, 85% in Switzerland, 79% in Hungary, 27% in Poland, and 9.9% in Belarus in 2009 (Unemo, 2015). At present, strains have been isolated that exhibit resistance to extended-spectrum cephalosporins (including ceftriaxone), which are the last drugs available for gonorrhea monotherapy. The detections of isolates with decreased susceptibility to cephalosporins (especially ceftriaxone) has been reported from all over the world (Unemo and Shafer, 2014). On average, the proportion of strains with reduced susceptibility to cefixime (MIC > 0.125 mg/L) in the European Union is approximately 8% (Unemo, 2015).

The reduced susceptibility mechanism is dependent on the mosaic organization of the *penA* gene, which may result from genetic recombination between the *N. gonorrhoeae, N. cinera*, and *N. perflava* species (Ohnishi et al., 2011). The resistance of *N. gonorrhoeae* (especially broad drug resistance to penicillins and cephalosporins) is associated with the emergence of mosaic alleles of *penA* and non-mosaic alleles carrying the A501 mutation. Mosaic alleles contain up to 70 amino acid substitutions compared to the wild-type protein. The mutations G545S, I312M, and V316T were found in strains resistant to broad spectrum cephalosporins. Their resistance can be explained by modification of the β-lactam binding site (Unemo et al., 2012; Lewis et al., 2013; Golparian et al., 2014). Notably, the introduction of these three mutations in the wild-type PBP2 has almost no effect on the resistance of the pathogen *N. gonorrhoeae*; these mutations enhance pathogen resistance only in the presence of other mosaic alleles in the *penA* gene. It is possible that this effect is due to epistasis (the suppression of a non-allelic gene) (Tomberg et al., 2010).

Reduced susceptibility of the gonorrhea pathogen to ceftriaxone is also associated with mutations of codon 501 in non-mosaic alleles of *penA* (most often A501V but sometimes A501T). The A501 mutation is assumed to be specific for *N. gonorrhoeae* because it is not found among other *Neisseria*; this mutation possibly appeared spontaneously in gonococci under selective antibiotic pressure and was not acquired from another species (Unemo et al., 2012). A detailed study of mutations in the *penA* gene of *N. gonorrhoeae* leading to its resistance to third generation cephalosporins (cefixime and ceftriaxone) was published by Kubanova et al. (Kubanova et al., 2014). Increased resistance to ceftriaxone was observed in the presence of mutations at positions 346, 505, 511, 517, 543, 567, 575, and 576 of PBP2. The replacement of glycine with serine at position 543 results in a manifold increase in resistance to ceftriaxone.

The first highly resistant strain to ceftriaxone (H041; NG-MAST ST4220) was a sporadic case in Japan. Another strain with a similar level of resistance (F89; NG-MAST ST1407; and MLST ST7363) was first described in France and now has spread throughout the world. These strains cause unsuccessful attempts of patient treatment with cefixime and ceftriaxone (Unemo and Nicholas, 2012; Morita-Ishihara et al., 2014). The *N. gonorrhoeae* H041 and F89 strains are highly resistant to third generation cephalosporins with a minimal inhibitory concentration (MIC) of 2–4 mg/L. An investigation of the *N. gonorrhoeae* strain H041 revealed 13 additional amino acid substitutions in a mosaic allele X *penA* compared to the mosaic *penA* gene from the strain with intermediate-level cephalosporin resistance (specifically, the mutations A311V and T316S located near the β-lactam-binding pocket close to the active-site nucleophile Ser310 and the mutation Thr483 that may interact with the carboxylate of the β-lactam antibiotic) (Tomberg et al., 2013). Two cases of extended-spectrum cephalosporin-resistant *N. gonorrhoeae* were recently isolated from patients in South Africa. These strains were shown to belong to a multidrug-resistant gonococcal clone (MLST ST1901) that was associated with several cefixime treatment failures in Europe and North America (Lewis et al., 2013).

The resistance of *N. gonorrhoeae* to macrolides (azithromycin) is caused by the presence of the *erm* (erythromycin ribosome methylation) and *mef* (associated with the active efflux of antibiotics) genes. The substitution C2611T (Ng et al., 2002) in the 23S rRNA gene (the *rrl* gene) leads to the emergence of strains with moderate resistance, whereas A2059G (Katz et al., 2012; Unemo et al., 2014) leads to the emergence of highly resistant strains. In addition to the nucleotide substitutions themselves, resistance depends on the number of mutant alleles. Unemo et al. (2014) demonstrated that the presence of the C2611T substitution in all four alleles resulted in a high level of resistance, whereas strains with one mutant allele did not differ from the wild type.

*N. gonorrhoeae* becomes resistant to tetracycline either of the expression of a plasmid-encoded TetM protein or by a combination of three gene mutations: (i) the *mtrR* mutation, which results in overexpression of an nonspecific efflux pump (MtrC-MtrD-MtrE) that promotes the efflux of a range of hydrophobic agents and detergents; (ii) the *penB* determinant, which is a mutated porin IB that decreases the influx of tetracycline into the cell, and (iii) and mutations in the *rpsJ* gene that result in the amino acid substitution V57M in the ribosomal protein S10 (Nguyen et al., 2014). Although the combination of these mutations does not confer a level of tetracycline resistance as high as that observed with tetracycline-specific efflux pumps or the TetM determinant, the *mtrR*-*penB*-*rpsJ1* gene triad is highly effective and provides levels of resistance above those clinically achievable at the site of infection. In the same time, the *rpsJ* allele acts independently of other resistance factors and increases the MIC of tetracycline between three- and four-fold (Hu et al., 2005).

There are several determinants of *N. gonorrhoeae* resistance to spectinomycin that binds to the 30S ribosomal subunit and inhibits translation during elongation by blocking the EF-G-catalyzed translocation of peptidyl-tRNA from the A site to the P site. One of these determinants affects the segment formation of the spectinomycin binding site (*rrs* gene) by introducing one mutation in the 16S rRNA (G1064C, G1058C, or C1192U) (Galimand et al., 2000). A high-level spectinomycin-resistant (MIC > 1 mg/L) *N. gonorrhoeae* strain was isolated in Norway. The resistance determinant was a deletion of codon 27 (valine) and a K28E alteration in the ribosomal protein S5 (Unemo et al., 2013). Mutation T24P in the *rpsE* gene leading to spectinomycin resistance has also been described (Ilina et al., 2013). Generally, there are few examples of MIC-verified stains of *N. gonorrhoeae* with high-level resistance to spectinomycin (Kirkcaldy et al., 2013; Unemo, 2015). However, in Russia the proportion of spectinomycin-resistant isolates increased from 0.9 to 11.6% in a short period between 2009 and 2012 (Kubanova A. et al., 2014).

At the end of 80's, since the introduction of quinolones (fluoroquinolones) for the primary treatment of uncomplicated gonorrhea, most of the isolates of gonococci were found to be extremely susceptible to fluoroquinolones. Since the importance of antimicrobial susceptibility studies was understood, data concerning fluoroquinolone-resistant *N. gonorrhoeae* strains became available from all parts of the world (Knapp et al., 1997). In these studies, it was evident that the resistance toward fluoroquinolones, which is chromosomally mediated, develops in an incremental manner. The initial isolates which were less susceptible toward ciprofloxacin were found to have MIC values of 0.06 mg/L, which gradually increased to 1 mg/L (such strains being referred to as intermediate resistant) and later to as high as 16 mg/L (classified as resistant isolates). Strains with MIC > 4 mg/L were considered as high level resistance strains.

The main mechanism of resistance to fluoroquinolones is the decrease in the affinity of the drug for the DNA-enzyme complex. This decrease is caused by mutations in the *gyrA, gyrB, parC*, and *parE* genes in the topoisomerase polypeptide chains that form the “quinolone pocket.” The resistance of *N. gonorrhoeae* to fluoroquinolones is associated with mutations S91F and D95N/G in *gyrA* gene and D86N, S88P, and E91K in *parC* gene (Vereshchagin et al., 2004). Mutations in *parC* at positions 87, 104, and 131 have also been described. Isolates with high level resistance may carry several mutations in the *gyrA* and *parC* genes (Vereshchagin et al., 2004; Pottumarthy et al., 2006). The more recent (fourth generation) fluoquinolones are more active against strains with altered ParC, but are less effective against GyrA mutants, thus, these compounds will in theory, be active against some, but not all, ciprofloxacin-resistant gonococci (Patel et al., 2011).

Resistance of *N. gonorrhoeae* to different antibiotics also arises as the result of modifications in the protein that transports antibiotics across the outer membrane (the porin PorB). In *N. gonorrhoeae*, porins are encoded by the *porB* gene and have a molecular mass ranging from 34 to 38 kDa in different strains (Zeth et al., 2013). Protein molecules form three-dimensional structures that puncture the outer membrane. Analysis of the penicillin- and tetracycline-resistant strains of *N. gonorrhoeae* revealed the G120D, A121D, and G121K mutations in the porin protein. Further studies demonstrated that these mutations were important for the diffusion of antibiotics (Olesky et al., 2006). In the same work, Olesky et al. described the G120K, G120D, and A121D mutations in the gene encoding *N. gonorrhoeae* porins. These mutations in the porin genes are widely spread and have been reported in Russia (Ilina et al., 2008), Switzerland (Endimiani et al., 2014), and Canada (Thakur et al., 2014). Notably, mutations in the *porB* gene do not increase the resistance of *N. gonorrhoeae* to β-lactams in the absence of mutations in the *mtrR* gene (a transcriptional repressor of the *mtrCDE* operon). Thus, the increase in resistance is caused by combined effort of the MtrC-MtrD-MtrE pump and PorB1b, which decreases the concentration of the antibiotic in the periplasm of the microorganism (Veal et al., 2002; Olesky et al., 2006).

**Figure 2:**
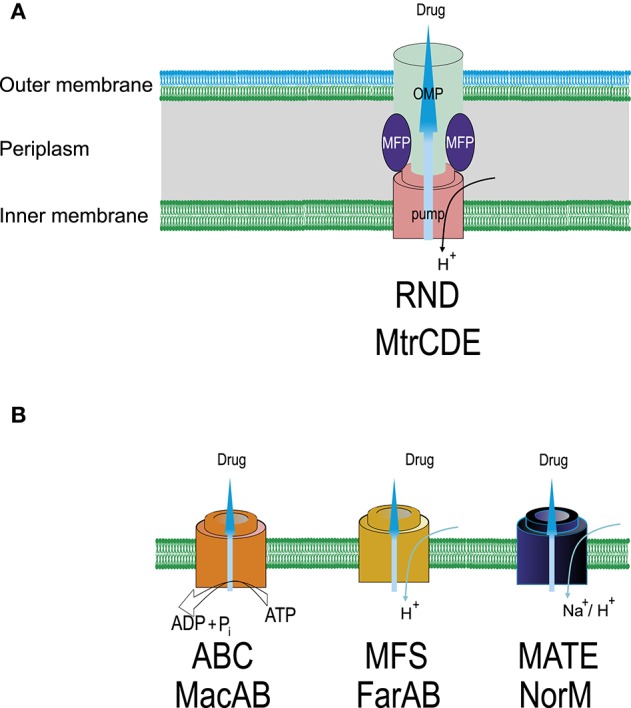
**Schematic representation of efflux systems found in ***N. gonorrhoeae***. (A)** MtrCDE, ternary efflux system crossing inner and outer membranes (RND superfamily). OMP, outer membrane channel protein; MFP, membrane fusion protein. Specific mutations in the promoter and/or coding sequence of *mtrR*—transcriptional repressor of the *mtrCDE* operon increase expression of the MtrCDE pump resulting in the increase in drug resistance, particularly, to ceftriaxone (*mtrR* mutations can also activate the *penB* resistance determinant). **(B)** Single-component transporters (ABC, MFS, MATE superfamilies). NorM is responsible for the efflux of fluoroquinolones, aminoglycosides, tetracyclines, FarAB—efflux of hydrophobic substances including macrolides. Overexpression of MacAB pump increases, mainly, MIC of macrolides. For the removal of antimicrobials ABC transporters use ATP as an energy source, other transporters employ an electrochemical gradient of cations, usually, protons.

Four efflux pump systems (MtrCDE, MacAB, NorM, and FarAB) have been found in gonococci belonging the RND, ABC, MATE, and MFS superfamilies (Alvarez-Ortega et al., 2013; Sun et al., 2014; Li et al., 2015; Sharkey et al., 2016), respectively (Figure 2). The most studied efflux pump from *N. gonorrhoeae* (MtrC-MtrD-MtrE from the RND superfamily) is responsible for the efflux of β-lactam antibiotics, macrolides, rifampin, detergents, fatty acids, steroid hormones, and cationic peptides (Veal et al., 2002). The efflux system involves a linker (MFP), a transporter, and a protein that forms a channel across the outer membrane (OMP protein). Inactivation of the MtrCDE pump in clinical isolates displaying high levels of MDR leads to significant decrease in the resistance to azithromycin, penicillin, and tetracycline (Golparian et al., 2014). Mutations in *mtrR* gene lead to the emergence of penicillin-resistant strains by increasing the expression of the MtrC-MtrD-MtrE efflux pump. Some of these mutations are located in the coding region and cause the amino acid substitutions A39T, R44H, G45D, and L47P (Ilina et al., 2008; Endimiani et al., 2014); others affect the promoter region (e.g., the insertions insTT at position −10 and deletion delA at position −35) (Lindberg et al., 2007; Ilina et al., 2008; Endimiani et al., 2014). The most common mutations are the deletion delA in the promoter region and the substitution G45D in the coding region of the gene. Other rarer mutations also exist, such as the insertion of 153 bp between the *mtrR/mtrC* promoter and the *mtrC* gene. The resistance of *N. gonorrhoeae* to fluoroquinolones is greatly increased by the G45D or −35 delA mutations (Zarantonelli et al., 1999).

The *macA* and *macB* genes for MacAB system are organized as an operon. A SNP in the promoter region increases the transcription of *macAB* and results in an increased resistance to macrolides (Rouquette-Loughlin et al., 2005). The Na/cation antiporter NorM belonging to MATE superfamily in *N. gonorrhoeae* removes fluoroquinolones, aminoglycosides, and cationic dyes. Inactivation of NorM results in an to eight-fold decrease in the MIC for tetracycline (Golparian et al., 2014). Mutation of the promoter region of the *norM* gene has been shown to reduce susceptibility to the fluoroquinolones ciprofloxacin and norfloxacin (Rouquette-Loughlin et al., 2003). In addition, the *mef* -encoded efflux pump protein, which is located on a conjugative transposon has been also found in *N. gonorrhoeae* strains. The protein exports macrolides out of the cell, thus, its overexpression results in resistance to macrolides (Luna et al., 2000). There is a synergistic interaction between the change in membrane permeability and the activity of efflux pumps. For example, simultaneous mutations in the porin protein and efflux pump from the RND superfamily in *N. gonorrhoeae* result in a significant increase in pathogen resistance to penicillin and ceftriaxone (Shafer and Folster, 2006).

As mentioned above, a multidrug-resistant strain from South Africa (Lewis et al., 2013) was resistant to cefixime, ciprofloxacin, penicillin, and tetracycline, exhibited intermediate resistance to azithromycin and was susceptible to ceftriaxone and the aminoglycosides, gentamicin and spectinomycin. The pathogen carried determinants of resistance for several antibiotics: mutations in the promoter region of *mtrR* gene (deletion A) resulting in overexpression of the MtrC-MtrD-MtrE efflux pump; G101K and A102N in *porB1b* encoding loop 3 of PorB1b, which decreases the influx of antibacterial drugs into the cell; mutation L421P in the *ponA1* gene that encodes the second penicillin target PBP1, thereby reducing the penicillin acylation of PBP1; and mosaic allele *penA*, type XXXIV, encoding the penicillin-binding protein PBP2.

Thus, *N. gonorrhoeae* has developed resistance to all antimicrobials introduced for first-line therapy during the last 70–80 years, and, probably, for more sustainable future treatment. To overcome this problem it is necessary to focus not only on derivatives of previously developed antimicrobials but also on the development and investigation of new targets, compounds, and approaches for treatment. As an example of such developments, the topoisomerase II inhibitor ETX0914 (also known as AZD0914) belonging to spiropyrimidinetriones was introduced as an antimicrobial compound. The mechanism of its action differs from the mechanisms of all previously and currently used drugs. The ETX0914 pharmacodynamic properties, antimicrobial susceptibility and genetic resistance mechanisms are now extensively studied for the future application as oral monotherapy or in dual combinations with ciprofloxacin, azithromycin, or ceftriaxon (Foerster et al., 2015).

### C. trachomatis

Information concerning the occurrence and clinical importance of *C. trachomatis* resistance to antibiotics is scarce and contradictory (Kohlhoff and Hammerschlag, 2015). Cases of multidrug resistance have been reported in *C. trachomatis* (Shkarupeta et al., 2007; Sandoz and Rockey, 2010). For example, failure of treatment with macrolides and doxycycline was reported in patients with chlamydial infection. Clinically isolated strains resistant to doxycycline, azithromycin, josamycin, spiramycin, and ofloxacin were reported, as were multidrug-resistant strains. Although certain molecular markers associated with resistance to macrolides and fluoroquinolones have been described, no functional relationship between the presence of these markers and the efficacy of antibiotic therapy for *Chlamydia* has been established. No mutations were found in the *gyrA, gyrB, parC*, and *parE* genes (resistance to fluoroquinolones) and in the 23S rRNA V-domain, which contains macrolide resistance-associated mutations in *C. trachomatis* clinical isolates obtained after ineffective therapy of urogenital chlamydiasis with fluoroquinolones and macrolides (Shkarupeta et al., 2007).

It is typical for chlamydia to acquire so-called heterotypic resistance, a form of phenotypic resistance in which a small proportion of an infecting microbial species is capable of expressing resistance at any one time. Strains isolated from patients after the failure of treatment with antimicrobial drugs are fully sensitive to the same drugs *in vitro*, and only a small fraction of the chlamydia population (< 1%) survives in the presence of high concentrations of the drugs (Shkarupeta et al., 2007).

### Mycoplasna and ureaplasma spp.

Because members of the genera *Mycoplasma* and *Ureaplasma* have no cell wall, they are insensitive to all types of β-lactam antibiotics (natural susceptibility). Sulfonamide at physiological concentrations also has no effect on these bacteria due to the absence of the metabolic pathway for the synthesis of folic acid. High rates of resistance to erythromycin and tetracycline in clinical specimens (from 73 to 97%) were reported for *Ureaplasma* species and *M. hominis.* Speciation indicated that *U. parvum* was the predominant *Ureaplasma* spp. that conferred antimicrobial resistance. Specimens resistant to macrolides were found, and cases of clinical and microbiological treatment failure with moxifloxacin were also reported (Redelinghuys et al., 2014). An increase in fluoroquinolone-resistant *M. genitalium* strains in Japan has been recently reported, and the prevalence of macrolide and fluoroquinolone resistance-associated mutations in DNA specimens from men with non-gonococcal urethritis has been determined (Kikuchi et al., 2014).

The resistance of *Mycoplasma* and *Ureaplasma* to antibiotics is primarily associated with mutations in the 23S rRNA (macrolides), *gyrA, gyrB, parC*, or *parE* gene (fluoroquinolones) (Table 1). The mutations responsible for macrolide, lincosamide, streptogramin, or ketolide group resistance occur in 23S rRNA at positions 2610, 2611, 2057, 2059, and 2062 in *M. hominis* (*Escherichia coli* numbering) and positions 2058 and 2059 in *M. genitalium*. Deletions and insertions in the L4 ribosomal proteins and mutations in the 23S rRNA at positions 2056, 2057, and 2058 are associated with macrolide resistance in *Ureaplasma* spp. (Couldwell et al., 2013; Salado-Rasmussen and Jensen, 2014; Waites and Xiao, 2015). The efflux genes and *erm* gene that contribute to the resistance of *Ureaplasma* to macrolides have been detected in only one work (Lu et al., 2010) and have not been confirmed by other investigators.

**Table 1:** **Molecular determinants of drug resistance and phenotypic susceptibility in Mycoplasmas and Ureaplasmas**.

**Antimicrobial drug**	**Determinants of drug resistance**	**Range of minimal inhibitory concentrations for resistant isolates (mg/L)**
***M. genitalium***		
Macrolides, lincosamides, streptogramins, ketolides	Mutations in the 23S rRNA gene at positions 2058 and 2059^*^	16–64 for erythromycin
	Mutations in L4 ribosomal protein	
Tetracyclines	No determinants have been detected for isolates obtained in cases of treatment failures	No data available
Fluoroquinolones	Mutations in the *gyrA, gyrB, parC*, or *parE* genes	No data available
***M. hominis***		
Macrolides, lincosamides, streptogramins, ketolides	Mutations in the 23S rRNA gene at positions 2610, 2611, 2057, 2059, and 2062^*^	16–64 for clindamycin
Tetracyclines	*tet*(M) determinant (ribosomal protection)	8–64 for tetracyclines
Fluoroquinolones	Mutations in the *gyrA, gyrB, parC*, or *parE* genes	2–32 for levofloxacin
		4–8 for ciprofloxacin
***U. urealyticum and U. parvum***		
Macrolides, lincosamides, streptogramins, ketolides	Deletions or insertions in L4 ribosomal proteins and/or mutations in the 23S rRNA gene at position 2056, 2057, and 2058	64–128 for erythromycin
	Ribosomal methylation mediated by the *ermB* gene	
	mrsA/mrsB/mrsD efflux pumps	
Tetracyclines	*tet*(M) determinant (ribosomal protection)	2–32 for tetracyclines
Fluoroquinolones	Mutations in the *gyrA, gyrB, parC*, or *parE* genes	2–16 for levofloxacin

* Escherichia coli numbering system.

Susceptibility of the *M. hominis* and *Ureaplasma* spp. to tetracyclines was studied in South Africa (Redelinghuys et al., 2014). The high-level resistance to tetracyclines in *M. hominis* and *Ureaplasma* spp. is explained by the presence of the *tet*(M) determinant that provides ribosome protection and represents the sole tetracycline resistance mechanism acquired by clinical isolates of human mycoplasmas (Dégrange et al., 2008). For *M. genitalium*, treatment failures with tetracycline were reported but no genes responsible for the resistance were identified (Waites and Xiao, 2015).

## Drug resistance of BV associated bacteria

### G. vaginalis

*G. vaginalis* is treated with metronidazole and clindamycin but only limited data are available with respect to its resistance (Nagaraja, 2008; Tomusiak et al., 2011). The putative mechanism for tetracycline resistance is the presence of the *tetM* gene, which is found in tetracycline-resistant strains of *G. vaginalis* (Harwich et al., 2010). Resistance to metronidazole has been found in some *G. vaginalis* strains. The proposed mechanisms of resistance include (Löfmark et al., 2010):

- A suppressed rate of activation of the drug inside the cell through its reduction;
- Increased activity of DNA repair systems;
- Increased activity of enzymes that consume oxygen (i.e., catalase, peroxidase, and superoxide reductase);
- Accelerated clearance of the drug from the cell by active efflux.

The most well-characterized mechanism of resistance to metronidazole is the inactivation or deletion of genes with nitroreductase activity (Dhand and Snydman, 2009). Some isolates have nitroreductase genes; however, the correlation between these genes and metronidazole resistance has not been studied.

Recent whole-genome sequencing studies have revealed that *G. vaginalis* has a population structure that consists of four clades: clades 1 and 3 are associated with bacterial vaginosis and clades 2 and 4 are not. Metronidazole susceptibility is associated with the population structure, with clade 3 and 4 isolates showing 100% resistance to this drug whereas the resistance of clade 1 and 2 are 35 and 7.1%, respectively (Schuyler et al., 2015b).

### B. fragilis

Recently, an increasing resistance to different antimicrobial drugs has been reported for *B. fragilis*. Resistant strains were discovered in both European countries and the United States. (Eitel et al., 2013). Multidrug-resistant *B. fragilis* isolates bearing parallel resistance to imipenem, amoxicillin and metronidazole or clindamycin were also found in Russia (Shilnikova and Dmitrieva, 2015). Antibiotic resistance is spread horizontally among the *B. fragilis* group of clinical isolates due to the antibiotic resistance genes carried on conjugative and mobilizable plasmids, conjugative transposons and integrated genetic elements (Eitel et al., 2013).

The most important mechanism of resistance of *B. fragilis* to β-lactam antibiotics is the production of β-lactamases (Edwards, 1997). The *cepA* gene encodes β-lactamase, which is able to destroy penicillins and most cephalosporins (except cefoxitin). Resistance to cefoxitin-resistant strains was explained by the presence of the *cfxA* gene located on the MTn4555 transposon. Resistance to carbapenems was explained by the presence of the *cfiA* gene in the bacterial chromosome. Because the *cfiA* gene may be expressed differently or even be silenced, different levels of carbapenem resistance may be detected. As recently shown (Eitel et al., 2013), the *cfxA* gene is not a major factor in determining cefoxitin resistance, *cfxA* was found with a higher prevalence in non-fragilis *Bacteroides* strains than in *B. fragilis*.

Metronidazole remains the drug of choice for the treatment of infections caused by *B. fragilis*, although metronidazole-resistant strains have been described in the literature (Brook, 2004). The resistance is due to the presence of the nitroimidazole resistance genes *nimA-nimG* on plasmids or on chromosomes (Carlier et al., 1997).

*B. fragilis* resistance to tetracycline originates from the presence of the *tetQ* gene (ribosome protection mechanism) and the *tetX* and *tetX1* genes encoding FAD-dependent monooxygenases that are able to destroy tetracycline.

*B. fragilis* resistance to clindamycin occurs via two mechanisms. First, the *ermB, ermF*, and *ermG* genes encoding various N^6^-methyltransferases modify the 23S RNA. Several transferable plasmids can cause resistance [pBF4, pBFTM10 (pCP1), and pB1136]. The *ermF* resistance gene was found on transposons Tn4351 (pBF4), Tn4400 (pBFTM10), and Tn4551 (pB1136). Among the possible clindamycin resistance genes, *ermF* was the most common and had the largest effect on clindamycin resistance after the *linA* gene. The second mechanism is the action of the efflux pumps encoded by the *msrSA* and *mefA* genes. The *msrSA* gene was first described for *Staphylococcus aureus* and was later also found in *Bacteroides.* The presence of the *ermG-mefA-msrSA* combination was confirmed for clindamycin-resistant *B. fragilis* strains (Eitel et al., 2013).

Members of the *Bacteroides* genus were originally resistant to first and second generation fluoroquinolones. Resistance to the next generation fluoroquinolones appeared in the last few years; however, fluoroquinolones of the third and fourth generations are still effective against *B. fragilis.* The BexA efflux pump encoded by the *bexA* gene may be responsible for the resistance of *B. fragilis* to fluoroquinolones and the elevated moxifloxacin MIC values. The BexA pump belongs to the MATE class. It was first described for *Bacteroides thetaiotaomicron*; disruption of BexA in *B. thetaiotaomicron* made the bacteria more susceptible to norfloxacin, ciprofloxacin, and ethidium bromide. The BexA protein sequence is homologous to the protein sequence of NorM, which is a multidrug efflux transporter of *Vibrio parahaemolyticus* (Miyamae et al., 2001).

### A. vaginae

Some studies demonstrated that *A. vaginae* could exhibit a high level of resistance to metronidazole (Geißdörfer et al., 2003). For example, several isolates of *A. vaginae* described in De Backer et al. (2006) were found to be highly resistant to metronidazole and susceptible to clindamycin, which are the two preferred antibiotics for the treatment of bacterial vaginosis; however, a large amount of variability in the susceptibility to metronidazole was reported (ranging from 2 to more than 256 mg/L). The authors concluded that metronidazole resistance was not an intrinsic feature of *A. vaginae.* Further research is required to clarify whether this metronidazole resistance might be acquired by the presence and activation of *nim*-genes encoding an alternative reductase that can convert nitroimidazole to a nontoxic derivative, thereby circumventing the toxic effect that causes breakage of the DNA (Löfmark et al., 2010).

A draft genome sequence of a metronidazole-susceptible (MIC 16 mg/L) vaginal isolate of *A. vaginae* (strain 44061) was recently published (Schuyler et al., 2015a). This information will be useful for comparative studies of the mechanism and the molecular basis of metronidazole resistance in *A. vaginae.*

### M. mulieris

In different studies, the resistance of *M. mulieris* to metronidazole varied from 50 to 81%. The MIC_90_ of metronidazole was 128 mg/L. Reports have suggested that 4% of *Mobiluncus* species are clindamycin-resistant (Spiegel, 1987; Bahar et al., 2005). A high prevalence of metronidazole-resistant *Mobiluncus* species (81%) was found among Turkish women with gynecological infections including bacterial vaginosis (Bahar et al., 2005). Notably, the *tetQ* gene that was associated with resistance to tetracycline was found in *Mobiluncus*.

## Conclusion

The emergence of resistance to antimicrobials is a natural consequence of the evolutionary process under increasing pressure from chemotherapy. As a result, resistance of STD pathogens appears to not only natural β-lactam antibiotics, but also to fully synthetic drugs such as fluoroquinolones. In the ongoing war between the disease and its effective treatment, a detailed understanding of the mechanisms of action of antibiotics and the emergence of resistance is necessary to modify existing drugs and to develop new ones directed against new targets. An ever-increasing range of reproductive tract bacterial infections calls for developing rapid, sensitive, and reliable methods that are able to determine resistance to available antimicrobials in individual patients as early as possible. At the same time, the recommended therapeutic regimen should take into account the regional occurrence of resistance to specific drugs obtained by constant monitoring. Moreover, for adequate therapy of diseases of the urogenital tract, in particular, for targeted selection of drugs, it is very important to take into account the possible polymicrobial character of a disease and to be able to simultaneously identify several STD pathogens and BV associated bacteria in individual clinical samples. These strategies will allow the use of existing drugs for a longer time with better efficiency and suppress the occurrence of multidrug-resistant bacterial microflora generated by repetitive failures of treatment with already inefficient antibiotics.

## Author contributions

BS and ED were the primary authors, conceived the study, and drafted the manuscript for publication. AR participated in writing Chapter Brief Description of the Most Common Bacterial Infection of the Human Reproductive System and the Drugs Used to Treat Them. AL wrote Section *T. pallidum*. DV, XP, and AK participated in writing Section *N. gonorrhoeae* of Chapter Drug Resistance of Std Pathogens. DD participated in writing Sections *C. trachomatis* and Mycoplasna and Ureaplasma spp. of Chapter Drug Resistance of Std Pathogens. DG wrote Chapter Drug Resistance of BV Associated Bacteria, and reviewed the initial and revised versions of the manuscript. All authors read and approved the final manuscript.

## Funding

This work was financed by subsidy #14.607.21.0065 (RFMEFI60714X0065) from the Ministry of Education and Science of the Russian Federation.

### Conflict of interest statement

The authors declare that the research was conducted in the absence of any commercial or financial relationships that could be construed as a potential conflict of interest.
